# Tobacco use among students aged 13–15 years in Greece: the GYTS project

**DOI:** 10.1186/1471-2458-7-3

**Published:** 2007-01-08

**Authors:** Athina Kyrlesi, Elpidoforos S Soteriades, Charles W Warren, Jeni Kremastinou, Panagiotis Papastergiou, Nathan R Jones, Christos Hadjichristodoulou

**Affiliations:** 1 Department of Public Health, Ministry of Health and Social Solidarity, Athens, Greece; 2 Department of Environmental Health, Environmental and Occupational Medicine and Epidemiology (EOME), Harvard School of Public Health, Boston, MA, USA; 3 Office for Smoking and Health, Centers for Disease Control and Prevention, Atlanta, Georgia, USA; 4 Department of Public Health, National School of Public Health, Athens, Greece; 5 Department of Hygiene and Epidemiology, Medical Faculty, University of Thessaly, Larisa, Greece

## Abstract

**Background:**

Data on the prevalence of tobacco use among teenagers in Greece are limited. We examined the prevalence of smoking among middle-school students in Greece using the Global Youth Tobacco Survey (GYTS).

**Methods:**

The Global Youth Tobacco Survey was implemented in Greece during the academic year 2004 – 2005 by the University of Thessaly and the National School of Public Health. Data were collected using the GYTS self-administered anonymous questionnaire, which was distributed by specifically trained field workers to a nationally representative sample of middle-school students aged 13–15 years (through randomly selected schools and classes), randomly selected through a two-stage cluster sample design. Data processing and statistical analyses were performed at the Centers for Disease Control and Prevention (CDC).

**Results:**

About one third of the students 32.1% (29.4 – 35.0) reported that they had tried tobacco in the past, while 16.2% (14.3 – 18.4) reported being current users of tobacco products. In addition, 1 in 4 of ever smokers reported that they began smoking before the age of 10 years old. Almost 1 in 5 never smokers reported being susceptible to initiate smoking in the next year and about 89.8% (88.3 – 91.1) of the respondents were exposed to environmental tobacco smoke in their homes and 94.1% (93.2 – 94.9) in public places. Finally, a strikingly high number of students 95% (89.5 – 97.7) reported that they were able to buy their own cigarettes without restrictions.

**Conclusion:**

The results of the GYTS show that the prevalence of smoking in middle-school children is alarmingly high in Greece. Smoking among young people constitutes a significant problem that is destined to worsen in the absence of any comprehensive efforts focused on strict anti-smoking legislation, policies and tobacco control interventions targeting children at a young age.

## Background

Tobacco use is one of the most important preventable causes of premature death, disease and disability around the world [1,2]. Nearly 5 million persons die annually from tobacco-related illnesses, and many more suffer from smoking-related morbidity while the number of fatalities is expected to more than double by year 2020 [3-5]. The Global Youth Tobacco Survey (GYTS), as part of the Global Tobacco Surveillance System (GTSS), initiated by the World Health Organization (WHO), the Centers for Disease Control and Prevention (CDC), and the Canadian Public Health Association (CPHA), was developed to monitor tobacco use, elicit attitudes about tobacco, and obtain information on exposure to tobacco smoke among youth. It has been completed so far by over 2 million students around the world [6].

Although, a few studies have been conducted in the past with respect to the prevalence of smoking among high-school students in Greece, there are no data available on smoking among middle-school students [7-9]. In addition, in contrast to most countries in the European Union (EU), where per capita consumption has decreased, the annual per capita consumption of cigarettes has risen steadily in Greece since the 1970s among adults and teenagers 15 years of age or older. In the mid '90s, annual per capita consumption in Greece was one of the highest in the EU; averaging about 4,300 cigarettes [10]. Therefore, the need to examine the problem of smoking among youth constitutes an urgent priority for Greece.

The objective of our current study was to examine the prevalence of smoking among middle-school students and to describe the prevalence of different indicators of smoking consumption using the information collected from the Global Youth Tobacco Survey (GYTS) implemented in Greece in 2005. We present data on the prevalence of different indicators of smoking including lifetime cigarette smoking, age of initiation, current smoking and tobacco dependency as well as current use of other tobacco products. Data are also presented on second-hand smoke exposure among students, and susceptibility of students to smoking.

## Methods

The Global Youth Tobacco Survey (GYTS) was implemented in Greece by the University of Thessaly, School of Medicine and the National School of Public Health in collaboration with the Ministry of Health and Social Solidarity.

### Study population

The Global Youth Tobacco Survey was administered to a representative sample of middle school students in Greece. We first divided the country into four major geographic regions taking into account the population density of each region (Athens, Thessalonica, Greek mainland and Islands) and using a two-stage cluster sample design in order to generate a representative sample of students 13 to 15 years of age. Our GYTS sampling frame included all schools containing the middle-school grades in Greece. A total of 100 schools were selected (25 schools from each region) in order to obtain a sample design that would produce representative, independent, cross-sectional estimates for each region. At the first stage, the probability of schools being selected was proportional to the number of students enrolled in the specified grades (grades 1–3 at all middle schools). At the second sampling stage, classes within the selected schools were randomly selected. All students in selected classes attending school on the day of the survey were eligible to participate.

### Data collection

The survey was approved by the Committee on Health Promotion of the Ministry of National Education and Religions. Parents were notified by a letter in advance of the survey and students gave verbal consent to complete the questionnaire. Students participated in the survey on a voluntary basis and data were collected anonymously using a self-administered questionnaire, which has been used around the world. The GYTS follows a standardized methodology for constructing sampling frames, selecting schools and classes, preparing questionnaires, carrying out field procedures, and processing data [11,12]. The questionnaires were distributed to each class of the selected schools during the school year 2004 – 2005, by field workers specifically trained for the GYTS project at the National School of Public Health during a day-long seminar.

The GYTS questionnaire included data on the prevalence of cigarette and other tobacco use, questions on perceptions and attitudes about tobacco, access and availability of tobacco products, susceptibility to initiate smoking, exposure to second-hand smoke, school curricula, media and advertising, smoking cessation as well as some demographic information, thereby providing a systematic approach for the surveillance of youth tobacco use among students. A project coordinator supervised the entire data collection process and reported to the principal investigator on a daily basis. Students' responses were documented on standardized answer sheets and sent to the Centers for Disease Control and Prevention for further processing where they were transformed into electronic files.

### Statistical analyses

Our analyses were performed using SUDAAN, a software package for statistical analysis of correlated data, in order to compute standard errors of the estimates and 95% confidence intervals [13]. A weighting factor was applied to each student record to adjust for non-response (by school, class, and student) and variation in the probability of selection at the school, class, and student levels. A final adjustment summing up the weights by grade and gender to the population of school children in the selected grades in each sample site was also taken into account. Differences were considered statistically significant at the p < 0.05 level and were two sided for all tests.

## Results

### Prevalence

In total, 6,378 students completed the GYTS. The school response rate was 90%, while the student response rate was 89%, and the overall response rate (i.e., the school rate multiplied by student rate) was 80%. The results of our analysis showed that 32.2% of 13–15 year old students in Greece had ever smoked (Table 1) and approximately 1 in 4 ever smokers of both genders initiated smoking before the age of 10. Slightly more than 1 in 10 students were current smokers of cigarettes (10.4%) or used other tobacco products (10.9%); however there was no statistical difference in tobacco use among boys and girls. Results also showed that 10.5% of current smokers wanted to have a cigarette within 30 minutes of waking up each morning and that 19.5% of never smokers indicated they were likely to initiate smoking in the next year, with no significant difference between boys and girls. For both genders the proportion of never smokers being susceptible to initiate smoking (19.4% for both genders) was significantly higher than the current smoking rate (11.8% for boys and 8.9% for girls).

**Table 1:** Prevalence of tobacco use among students aged 13–15 years, by gender – Greece, 2005

**Smoking Prevalence**	**Prevalence (95% Confidence Intervals)**		
	**Boys**	**Girls**	**Total**
Ever smoked cigarettes	34.6 (30.8 – 38.7)	28.9 (26.4 – 31.5)	32.1 (29.4 – 35.0)
Smoked cigarettes before age 10	25.5 (21.8 – 29.5)	21.4 (17.1 – 26.5)	23.3 (20.2 – 26.6)
Current cigarette smoker	11.3 (9.4 – 13.6)	9.0 (7.2 – 11.3)	10.4 (8.8 – 12.4)
Current user of tobacco products other than cigarettes	11.8 (10.1 – 13.8)	8.9 (7.2 – 11.0)	10.9 (9.4 – 12.5)
Current user of any tobacco product *	17.1 (15.0 – 19.4)	14.4 (12.1 – 16.9)	16.2 (14.3 – 18.4)
Current smokers dependent on tobacco †	8.8 (4.5 – 16.5)	10.9 (5.9 – 19.2)	10.5 (7.2 – 15.1)
Students susceptible to initiate smoking in the next year	19.4 (17.1 – 22.0)	19.4 (17.0 – 22.1)	19.5 (17.5 – 21.7) §

* Anyone who is a current cigarette smoker or current user of other tobacco products

† Students reported that they wanted to have first cigarette less than 30 minutes after waking up

§ Total includes some students that did not provide information on the gender question

### Secondhand smoke

Exposure to secondhand smoke as presented in Table 2 is extremely high in Greece; both at home (89.8%) and in public places (94.1%). Exposure in the home and in public is high for both never smokers and current smokers (over 9 in 10 students). In addition, never smokers (90.1%) were significantly more likely than current smokers (53.9%) to favor a ban on smoking in pubic places.

**Table 2:** Prevalence of exposure to secondhand smoke and support for a ban on smoking in public places among students aged 13–15 years, by smoking status – Greece, 2005

**Second-hand Smoke**	**Prevalence (95% Confidence Intervals)**		
	**Never Smokers**	**Current Smokers**	**Total**
Students exposed to smoke at home	88.0 (86.3 – 89.4)	93.6 (89.7 – 96.2)	89.8 (88.3 – 91.1)
Students exposed to smoke in public places	93.4 (92.2 – 94.5)	96.1 (93.5 – 97.7)	94.1 (93.2 – 94.9)
Students who support ban on smoking in public places	90.1 (88.4 – 91.6)	53.9 (48.1 – 59.6)	84.8 (82.7 – 86.6)

### Media and advertising

Almost 9 in 10 students (89.4%) reported seeing an anti-smoking media message during a period of 30 days prior to the survey (Table 3). However, only half of the students (49.3%) reported seeing anti-smoking messages at sports or other community events. Furthermore, approximately 7 in 10 students reported being exposed to pro-cigarette advertisements on billboards (70.3%), in newspapers or magazines (75.6%), or at sporting or other community events (69.9%). Almost 2 in 10 students reported having an object (i.e., t-shirt, backpack, etc) with a cigarette brand logo on it, with boys (23.0%) significantly more likely than girls (15.8%) to have such an object. In addition, about 2 in 10 students (16.7%) reported ever being offered free cigarettes by a tobacco company representative.

**Table 3:** Tobacco control factors – Media and Advertising – as reported by students aged 13–15 years, by gender – Greece, 2005

**Media and Advertising**	**Prevalence (95% Confidence Intervals)**		
	**Boys**	**Girls**	**Total**
Saw anti-smoking media messages in the past 30 days	89.9 (88.5 – 91.2)	88.6 (86.8 – 90.2)	89.4 (88.2 – 90.4)
Saw anti-smoking messages at sporting or other events in the past 30 days	52.8 (49.6 – 55.9)	45.7 (42.8 – 48.5)	49.3 (47.0 – 51.7)
Saw pro-cigarette ads on billboards in the past 30 days	71.1 (67.2 – 74.7)	69.5 (65.5 – 73.3)	70.3 (67.1 – 73.4)
Saw pro-cigarette ads in newspapers or magazines in the past 30 days	74.4 (71.5 – 77.0)	76.7 (73.4 – 79.7)	75.6 (72.9 – 78.2)
Saw pro-cigarette messages at sporting or other events in the past 30 days	70.8 (68.6 – 73.0)	68.5 (65.6 – 71.4)	69.9 (67.8 – 71.9)
Have an object with a cigarette brand logo on it	23.0 (20.7 – 25.5)	15.8 (14.2 – 17.5)	19.6 (18.3 – 21.0)
Ever offered free cigarettes by a tobacco company representative	18.1 (16.3 – 20.0)	15.1 (13.5 – 16.8)	16.7 (15.3 – 18.1)

### Cessation

With respect to smoking cessation, almost 4 in 10 students, among current smokers (37.6%), reported that they wanted to stop smoking immediately (Table 4). Also, almost half of the student current smokers had tried to quit smoking during the past year, but failed to succeed. There were no gender differences comparing the desire to stop smoking or the reports of quit attempts.

**Table 4:** Tobacco control factors – Cessation, Access, and School Curricula – as reported by students aged 13–15 years, by gender – Greece, 2005

**Cessation – Current Smokers**	**Prevalence (95% Confidence Intervals)**		
	**Boys**	**Girls**	**Total**
Want to stop smoking now	37.5 (28.0 – 48.0)	37.2 (29.1 – 46.1)	37.6 (31.3 – 44.4) *
Tried to quit smoking in the past year	56.9 (48.4 – 65.0)	60.6 (49.7 – 70.5)	57.9 (50.6 – 64.9)
**Access – Current Smokers**			
Usually buy cigarettes in a store	52.5 (45.1 – 59.7)	43.1 (35.2 – 51.5)	49.1 (43.0 – 55.3)
Not refused purchase because of their age	94.7 (86.5 – 98.0)	97.8 (89.4 – 99.6)	95.0 (89.5 – 97.7)
**School Curricula**			
Taught in class about the dangers of smoking	63.5 (60.0 – 66.9)	66.1 (2.13 – 69.9)	64.7 (61.3 – 67.9)
Discussed in class the reasons why people their age smoke	48.5 (44.2 – 52.8)	37.9 (32.8 – 43.2)	43.5 (39.4 – 47.8)

* Total includes some students that did not provide information on the gender question

### Access

Finally, questions on access to cigarettes for underage students showed that about half of current smokers (49.1%) reported that they usually bought their cigarettes by purchasing them from a store (Table 4). Most importantly, over 9 in 10 student smokers (95.0%), who purchased cigarettes from a store, were not refused purchase because of their age. In addition, there was no gender difference comparing the places where smokers usually got their cigarettes or in the students' ability to purchase cigarettes from a store. Finally, over 6 in 10 students (64.7%) reported being taught in school during the past year about the dangers of smoking (Table 4). However, only 4 in 10 students discussed in class the reasons why young people choose to smoke.

## Discussion

Our study is one of the first comprehensive evaluations of the problem of smoking among adolescent students in Greece. We found a relatively high prevalence of current smokers among middle-school students for both cigarettes and other tobacco products. In addition, students were susceptible to initiate smoking in the next year at twice the prevalence of current users. An important finding included the very high percentage of students being exposed to second-hand smoke in both their home as well as in public places. Another disturbing finding was also the fact that girls were as likely as boys to smoke cigarettes and also use other forms of tobacco.

Findings in this report indicate that tobacco control efforts in Greece face a number of significant challenges. First, the finding that boys and girls smoke at a comparable level is in contrast with previous studies among high-school students [7,8], where the smoking prevalence was higher among boys compared with girls. Furthermore, boys and girls, who have never smoked, do not differ in their likelihood to initiate smoking in the near future. Second, the prevalence of susceptibility for smoking initiation among never smokers (19.5%) was almost twice that of current cigarette smokers (10.5%), for both genders, possibly suggesting that adolescents smoking rates are likely to increase dramatically in the next few years. Third, the prevalence of tobacco use other than cigarettes (10.9%) is similar to that of cigarettes (10.4%), indicating that tobacco control efforts in Greece should address all forms of tobacco products and not just cigarettes. Fourth, the high rate of students' exposure to second-hand smoke indicates a need to enforce already existing laws governing smoking and exposure to second-hand smoke in public places. Creating smoke-free areas and educating the public about the dangers of second-hand smoke will have complementary effects on efforts to control smoking by reducing the social acceptability of tobacco use around people who do not smoke especially children [14,15]. Moreover an alarming finding of our study is the age of smoking initiation, which is decreasing over time with 1 in 4 students initiate smoking at the age of 10 years old. Such finding suggests that the anti-smoking campaign should also target students of elementary school and especially the fifth and sixth grades.

Greece is one of the first European countries to implement the Global Youth Tobacco Survey (GYTS). Previous limited reports showed that Greece had one of the highest smoking rates. However, few European countries have implemented GYTS and have reported smoking rates among their middle-school students. For example, GYTS in Hungary in 2003 showed that 33.5% of students report current use of cigarettes, whereas in Slovenia the percentage was 28.5% and in Slovakia the same survey showed that 24.3% were current users of cigarettes as opposed to only 10.4% in Greece. The corresponding rate of current cigarette use in Poland in 2003 was 23.3%, in Romania it was 23.2% and in Albania it was only 12%, which was very similar to Greece. Therefore, the results show that there is a much lower cigarette use among middle-school students in the Balkans as opposed to central Europe.

Several laws regulate the advertisement and sale of tobacco products in Greece. However, one of the weaknesses of the current situation is the efforts to enforce the existing legislation. For example, the European Union legislation on labeling, requiring rotating health warnings and listing of tar and nicotine levels, has also been implemented into Greek law. However, there are no laws regulating sales to minors, although vending and single stick sales of cigarettes are banned and the sale of tobaccoproducts through automatic vending machines is prohibited. A strong indication for the need to develop and implement legislation on the access of cigarette products for minors is the extremely high percentage of students being able to buy cigarettes from stores (95%). In addition, although new legislation has been introduced restricting smoking in government buildings, hospitals, trains, buses, and other public places, there is an urgent need for law enforcement in order to protect both adults and children from second-hand smoke.

A number of limitations of our study need to be acknowledged. First, there was some non-response in our study that may have introduced selection bias if the non responding was associated in any way with smoking. In addition, the possibility of information bias cannot be excluded given that some students may not provide valid answers and also may underreport the level of smoking since the data collected are based on a self-administered questionnaire. Nevertheless, in our study, we reached a student participation rate, which although not perfect, it was sufficient enough to draw significant conclusions on several scientific questions [16]. Furthermore, another limitation was that we used several field workers, however we do not believe that this introduced bias in the data collection process since the questionnaire was self-administered by the students. Although, GYTS represents a cross-sectional survey, it is an excellent tool for Greece to track changes over time and evaluate future tobacco control programs. The Ministry of Health and Social Solidarity in Greece has made initial efforts in tobacco control by establishing a tobacco control unit and making tobacco control a priority among health care workers and youth, however many more programs are needed to considerably improve the current situation.

The systematic surveillance of youth tobacco use constitutes an essential first step in our efforts to prevent the worldwide epidemic of death, disease and disability that smoking is projected to cause in the 21^st ^century. An important finding, revealed by our study, is the level of exposure of a large proportion of students to second-hand smoke, which needs to be addressed in a systematic way because it affects not only the smokers but almost all students at home and in public places. The results from the first GYTS implemented in Greece have significantly enhanced our capacity to develop, implement, and evaluate tobacco prevention and control programs in Greece.

## Conclusion

The Ministry of Health and Social Solidarity in Greece in collaboration with public and private agencies is urgently called upon to use the current baseline data in order to develop a comprehensive tobacco control program targeting children and adolescent smoking rates as well as the issue of passive smoking. Future repeated rounds of the GYTS should also be used to evaluate the effectiveness of recommended programs at consecutive intervals. The problem of smoking in Greece has now been documented in its most alarming dimensions, and there is no excuse to stay still while the tobacco industry continues to target our children on a daily basis.

## Competing interests

The author(s) declare that they have no competing interests.

## Authors' contributions

AK, ESS, CWW, NRJ, JK and CH contributed to the design and development of the methodology of the study. ESS, CWW, NRJ and CH developed the questionnaire, while AK, ESS, PP, JK and CH contributed to, and supervised the data collection process. CWW, NRJ and ESS analyzed and interpreted the data. AK, ESS, CWW and NRJ developed the first draft of the manuscript. All authors contributed to the final version of the manuscript. All authors read and approved the final manuscript.

## Pre-publication history

The pre-publication history for this paper can be accessed here:



## References

[B1] Davis RM, Smith R (1991). Addressing the most important preventable cause of death. BMJ.

[B2] Novick LF (2000). Smoking is the leading preventable cause of death and disability in the United States. J Public Health Manag Pract.

[B3] Peto R, Lopez AD, Koop CE, Pearson CE, Schwarz MR (2001). Future worldwide health effects of current smoking patterns. Critical Issues in Global Health.

[B4] Wen CP, Tsai SP, Chen CJ, Cheng TY, Tsai MC, Levy DT (2005). Smoking attributable mortality for Taiwan and its projection to 2020 under different smoking scenarios. Tob Control.

[B5] Warren CW, Jones NR, Eriksen MP, Asma S, Global Tobacco Surveillance System (GTSS) collaborative group (2006). Patterns of global tobacco use in young people and implications for future chronic disease burden in adults. Lancet.

[B6] Global Youth Tobacco Survey Collaborating Group (2003). Differences in worldwide tobacco use by gender: findings from the Global Youth Tobacco Survey. J Sch Health.

[B7] Kokkevi A, Stefanis C (1991). The epidemiology of licit and illicit substance use among high school students in Greece. Am J Public Health.

[B8] Sichletidis LT, Chloros D, Tsiotsios I, Kottakis I, Kaiafa O, Kaouri S, Karamanlidis A, Kalkanis D, Posporelis S (2006). High prevalence of smoking in Northern Greece. Prim Care Respir J.

[B9] Koumi I, Tsiantis J (2001). Smoking trends in adolescence: report on a Greek school-based, peer-led intervention aimed at prevention. Health Promot Int.

[B10] World Health Organization Regional Office for Europe The European Report on Tobacco Control Policy, Review of implementation of the Third Action Plan for a Tobacco-free Europe 1997–2001.

[B11] Global Tobacco Surveillance System Collaborating Group (2005). Global Tobacco Surveillance System (GTSS): purpose, production, and potential. J Sch Health.

[B12] The GTSS Collaborative Group (2006). The global tobacco surveillance system. Tob Control.

[B13] Shah BV, Barnwell BG, Bieler GS (1997). Software for the statistical analysis of correlated data (SUDAAN). User's manual, release 75, 1997 [software documentation].

[B14] Pierce JP, Choi WS, Gilpin EA, Farkas AJ, Merritt RK (1996). Validation of susceptibility as a predictor of which adolescents take up smoking in the United States. Health Psychol.

[B15] Fichtenberg CM, Glantz SA (2002). Effects of smoke-free workplaces on smoking behavior: systematic review. BMJ.

[B16] Brener ND, Collins JL, Kann L, Warren CW, Williams BI (1995). Reliability of the Youth Risk Behavior Survey Questionnaire. Am J Epidemiol.

